# ﻿Three new species of the genus *Olios* Walckenaer, 1837 (Araneae, Sparassidae) from southern China

**DOI:** 10.3897/zookeys.1245.144552

**Published:** 2025-07-16

**Authors:** He Zhang, Weitao Niu, Jie Liu, Caijun Wang, Changhao Hu

**Affiliations:** 1 College of Physics and Electronic Engineering, Xingtai University, Xingtai 054001, Hebei, China; 2 Xingtai Innovation Center for Sweet Potato Germplasm and Rapid Propagation Technology, Xingtai 054001, Hebei, China; 3 College of Chemical Engineering and Biotechnology, Xingtai University, Xingtai 054001, Hebei, China; 4 Arachnid Resource Centre of Hubei Province & Hubei Key Laboratory of Regional Development and Environmental Response, Faculty of Resources and Environmental Science, Hubei University, Wuhan 430062, Hubei, China; 5 Centre for Behavioural Ecology and Evolution, School of Life Sciences, Hubei University, Wuhan 430062, Hubei, China; 6 Hubei Broad Nature Technology Service Co., Ltd., Wuhan 430079, Hubei, China

**Keywords:** Biodiversity, huntsman spiders, morphology, new division, sparassids, taxonomy

## Abstract

Three new species of the sparassid spider genus *Olios* Walckenaer, 1837 are described from China: *O.biprocessus* Hu, Zhang & Liu, **sp. nov.** (♂) from Hunan Province, *O.uniprocessus* Hu, Zhang & Liu, **sp. nov.** (♂) from Chongqing Municipality and *O.lincangensis* Hu, Zhang & Liu, **sp. nov.** (♂♀) from Yunnan Province. Diagnoses, descriptions, photos and a distribution map of these three new species are provided.

## ﻿Introduction

The genus *Olios* Walckenaer, 1837 is the third-largest genus in the spider family Sparassidae Bertkau, 1872, with 166 known species (World Spider Catalog 2025). For a long time, this genus was used as a “dumping ground” for the family Sparassidae (Jäger and Kunz 2005). More than 60 species previously assigned to *Olios* have been transferred to other genera in past studies, particularly among Neotropical species (e.g., *Caayguara* Rheims, 2010, *Curicaberis* Rheims, 2015, *Meri* Rheims & Jäger, 2022, *Nolavia* Kammerer, 2006, and *Sadala* Simon, 1880) (Rheims 2010, 2015; Jäger 2020; Rheims and Jäger 2022). Jäger (2020) further revised *Olios* and reported eight *Olios* species-groups (*argelasius*-group, *auricomis*-group, *coenobitus*-group, *correvoni*-group, *hirtus*-group, *nentwigi*-group, *rossettii*-group, and *stimulator*-group), comprising 71 species. However, many species remain improperly assigned to *Olios*, necessitating further systematic revision (Jäger 2020).

Phylogenetic analyses of Sparassidae have consistently demonstrated that *Olios* is a polyphyletic assemblage (Jäger and Kunz 2005; Moradmand et al. 2014; Tong et al. 2018; Gorneau et al. 2022), which may explain the remarkable morphological diversity of copulatory organs in *Olios* species. Recent phylogenetic work by Gorneau et al. (2022) delimited the scope of true *Olios*, which is a monophyletic clade represented by four species: *O.argelasius* (Walckenaer, 1806) (type species) from the Mediterranean, *O.rossettii* (Leardi, 1901) from southern Asia, *O.lamarcki* (Latreille, 1806) from Sri Lanka, and an unidentified species from Singapore.

In China, seven *Olios* species are currently known, which are classified into three species-groups: *O.menghaiensis* (Wang & Zhang, 1990) within the *hirtus*-group; *O.digitatus* Sun, Li & Zhang, 2011, *O.nanningensis* (Hu & Ru, 1988), *O.scalptor* Jäger & Ono, 2001 and *O.suung* Jäger, 2012 within the *nentwigi*-group; and *O.tiantongensis* (Zhang & Kim, 1996), *O.sericeus* (Kroneberg, 1875) within the *rossettii*-group (Kroneberg 1875; Hu and Ru 1988; Wang and Zhang 1990; Zhang and Kim 1996; Jäger and Ono 2001; Jäger and Yin 2001; Sun et al. 2011; Jäger 2012, 2020; Hu et al. 2025). The aim of the current paper is to contribute to the taxonomic knowledge of *Olios* in China by describing three new species.

## ﻿Material and methods

The specimens examined in this study are deposited in the Centre for Behavioral Ecology and Evolution (CBEE), College of Life Sciences, Hubei University in Wuhan. Specimens were examined using an Olympus SZX7 stereo microscope. Photographs were taken on a Leica M205C stereo microscope. The male palp was examined and photographed after dissection. The epigyne was examined after being dissected from the body and treated in a warm 0.1 mg/ml Protease K solution. Eye diameters were taken at the widest point. Legs and palpal measurements were given as total length (femur, patella, tibia, metatarsus [absent in palp], tarsus). All measurements were in millimeters (mm). Spination follows that given in Davies (1994).

The terminology used in this paper follows Jäger (2020). Abbreviations: **ALE** = anterior lateral eyes, **AME** = anterior median eyes, **C** = conductor, **CD** = copulatory duct, **CH** = clypeus height, **CO** = copulatory opening, **dRTA** = dorsal part of retrolateral tibial apophysis, **DS** = dorsal shield of prosoma, **E** = embolus, **ES** = epigynal slit, **FD** = fertilization duct, **Fe** = femur, **LL** = lateral lobes, **Mt** = metatarsus, **OS** = opisthosoma, **Pa** = patella, **PLE** = posterior lateral eyes, **PME** = posterior median eyes, **Pp** = palp, **ptA** = proximal tibial apophysis, **RTA** = retrolateral tibial apophysis, **S** = spermathecae, **TA** = tegular apophysis, **TAP I–IV** = projection I to IV of tegular apophysis, **Ti** = tibia, **vRTA** = ventral part of retrolateral tibial apophysis, **I, II, III, IV** = legs I to IV.

## ﻿Result

### ﻿Taxonomy

**Family Sparassidae Bertkau, 1872**

#### ﻿Genus Olios

Taxon classificationAnimaliaAraneaeSparassidae

Walckenaer, 1837

0046FFC5-3C5C-58BD-A5C3-7174BCF5CABE

##### Type species.

*Oliosargelasius* (Walckenaer, 1806).

##### Diagnosis.

See Jäger (2020).

***Oliosrossettii* -group**

**Diagnosis.** See Jäger (2020).

**Species included.***Oliosbaulnyi* (Simon, 1874), *O.bhattacharjeei* (Saha & Raychaudhuri, 2007), *O.biprocessus* Hu, Zhang & Liu, sp. nov., *O.brachycephalus* Lawrence, 1938, *O.floweri* Lessert, 1921, *O.jaldaparaensis* Saha & Raychaudhuri, 2007, *O.japonicus* Jäger & Ono, 2000, *O.kiranae* Sethi & Tikader, 1988, *O.kolosvaryi* (Caporiacco, 1947), *O.longipes* (Simon, 1884), *O.lutescens* (Thorell, 1894), *O.mahabangkawitus* Barrion & Litsinger, 1995, *O.obesulus* (Pocock, 1901), *O.rossettii* (Leardi, 1901), *O.rotundiceps* (Pocock, 1901), *O.sericeus* (Kroneberg, 1875), *O.sherwoodi* Lessert, 1929, *O.suavis* (O. Pickard-Cambridge, 1876), *O.tarandus* (Simon, 1897), *O.tener* (Thorell, 1891), *O.tiantongensis* (Zhang & Kim, 1996), *O.uniprocessus* Hu, Zhang & Liu, sp. nov.

**Remarks.** This species-group is characterized by the presence of one or two apophyses on palpal tibia, a variable tegular apophysis, and a small U-shaped embolus arising from the tegulum at the central to sub-central position (Jäger 2020). The two newly described species, *Oliosbiprocessus* Hu, Zhang & Liu, sp. nov. and *O.uniprocessus* Hu, Zhang & Liu, sp. nov., exhibit all the diagnostic characteristics of this species-group. Therefore, they clearly belong to this species-group.

#### ﻿ Olios biprocessus

Taxon classificationAnimaliaAraneaeSparassidae

Hu, Zhang & Liu sp. nov.

FE33BE0D-B156-5675-982A-9C2AFECF4EFF

https://zoobank.org/6525BA3E-BE51-4564-B1DD-5EA95D1F256A

Figs 1 , 2 , 8

##### Type material.

***Holotype*** male: China • Hunan Province: Changsha City, Yuelu District, Xihu Park, under bark, 28.20758°N, 112.94004°E, elevation 30 m, 19 December 2023 as subadult, molted to adult on 18 February 2024, Jiwei Lin leg.

##### Etymology.

The specific name is a combination of the Latin *bi* (meaning double) and *processus* (meaning process, projection), referring to the two projections (TAP III, TAP IV) on the embolic base; noun.

##### Diagnosis.

**Figure 1.**
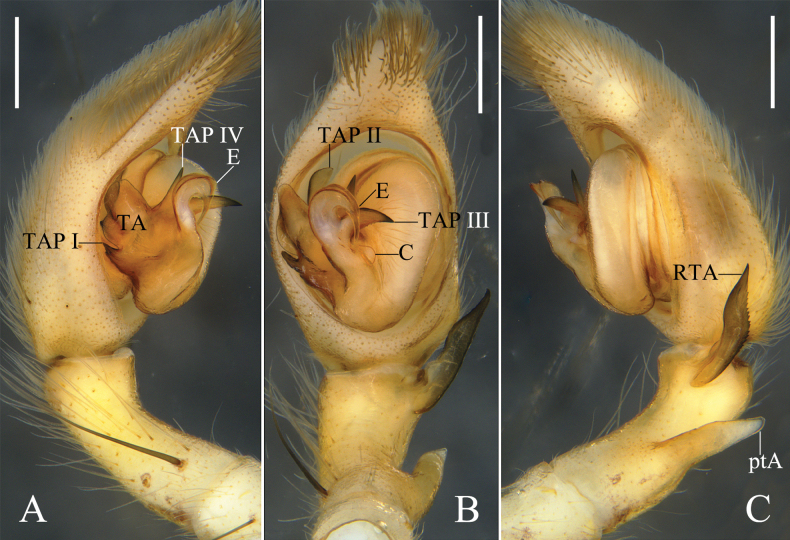
*Oliosbiprocessus* Hu, Zhang & Liu, sp. nov., left male palp. **A.** Prolateral; **B.** Ventral; **C.** Retrolateral. Abbreviations: C = conductor, E = embolus, ptA = proximal tibial apophysis, RTA = retrolateral tibial apophysis, TA = tegular apophysis, TAP I–IV = projection I to IV of tegular apophysis. Scale bars: 0.5 mm.

The male of *Oliosbiprocessus* Hu, Zhang & Liu, sp. nov. resembles that of *O.rossettii* (Leardi, 1901) (cf. Fig. 1A–C vs. figs 146, 147 in Jäger 2020) by having two tibial apophyses, and a small U-shaped embolus arising sub-centrally from the tegulum, but can be recognized by: 1) retrolateral tibial apophysis leaf-shaped in retrolateral view, with a serrated retrolateral margin; and 2) tegular apophysis complex, with a sharp projection (TAP I) prolaterally, and two sharp projections (TAP III, TAP IV) on the embolic base (vs. retrolateral tibial apophysis triangular, tegular apophysis long triangular in *O.rossettii*).

The male of *Oliosbiprocessus* Hu, Zhang & Liu, sp. nov. also resembles that of *O.uniprocessus* Hu, Zhang & Liu, sp. nov. (cf. Fig. 1A–C vs. fig. 3A–C) by having a horn-shaped proximal tibial apophysis, a leaf-shaped retrolateral tibial apophysis with a serrated retrolateral margin, sharp TAP I, lamellar TAP II, and a small U-shaped embolus arising sub-centrally from the tegulum, but can be recognized by the tegular apophysis with two sharp projections (TAP III, TAP IV) on the embolic base (vs. one projection (TAP III) in *O.uniprocessus* Hu, Zhang & Liu, sp. nov.).

##### Description.

**Male (holotype)**: Total length 8.5; DS 4.0 long, 4.1 wide; OS 4.6 long, 3.2 wide. Eyes: AME 0.24; ALE 0.19; PME 0.19; PLE 0.20; AME–AME 0.17; AME–ALE 0.17; PME–PME 0.42; PME–PLE 0.31; AME–PME 0.15; ALE–PLE 0.13; CHAME 0.18; CHALE 0.25. Measurements of palp and legs: Pp: 5.3 (1.5, 0.6, 1.1, -, 2.1); I: 19.8 (5.4, 1.6, 5.2, 6.0, 1.6); II: 20.3 (5.4, 1.9, 6.6, 3.1, 3.3); III: 14.9 (4.7, 1.3, 3.6, 3.6, 1.7); IV: 17.3 (5.3, 1.2, 4.6, 4.9, 1.3). Leg formula: II-I-IV-III. Spination: Pp: 131, 000, 2000; legs: Fe I–II 223, III 323, IV 321; Pa I–IV 000; Ti I 2124, II 2024, III 2124, IV 1204; Mt I–IV 2024. Cheliceral furrow with 2 anterior and 5 posterior teeth.

Palp (Fig. 1A–C): As in diagnosis, ptA arising submedially from Ti, RTA arising distally from Ti. Cymbium almost 1.5 times longer than Ti. TA complicated, with a sharp projection (TAP I) on prolateral margin, a lamellar projection (TAP II) on dorsal TA, and two sharp projections (TAP III, TAP IV) on embolic base. C tiny. E arising sub-centrally from tegulum in 9-o’clock-position.

**Figure 2.**
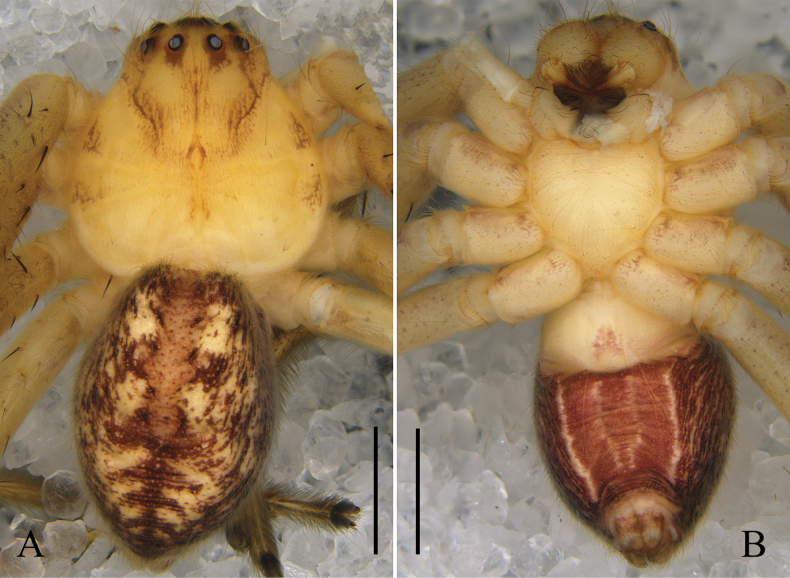
*Oliosbiprocessus* Hu, Zhang & Liu, sp. nov., male habitus. **A.** Dorsal; **B.** Ventral. Scale bars: 2 mm.

Colouration (Fig. 2A, B): DS pale yellow, with brown patterns. Sternum light yellow. Legs yellow to light brown, with brown spots. OS dorsally yellow, with yellowish-brown patterns. OS ventrally brownish purple, with four yellow longitudinal lines; anterior part yellow.

**Female**: Unknown.

##### Distribution.

China (Hunan Province) (Fig. 8).

#### ﻿ Olios uniprocessus

Taxon classificationAnimaliaAraneaeSparassidae

Hu, Zhang & Liu sp. nov.

4F1D16F8-C762-54A0-B67B-763E482E838E

https://zoobank.org/5F787A9C-980C-46CF-9ADB-0E9D9B5E9434

Figs 3 , 4 , 8

##### Type material.

***Holotype*** male: China • Chongqing Municipality: Beibei District, Southwest University, next to the international student apartment, 29.82852°N, 106.42972°E, elevation 253 m, 30 June 2015, Mingxin Liu leg.

##### Etymology.

The specific name is a combination of the Latin *uni* (meaning single) and *processus* (meaning process, projection), referring to the single projection (TAP III) on the embolic base, noun.

##### Diagnosis.

**Figure 3.**
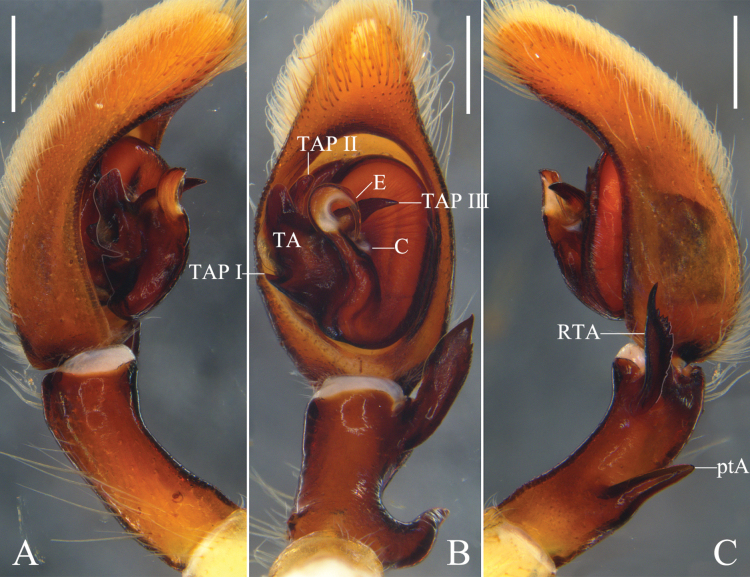
*Oliosuniprocessus* Hu, Zhang & Liu, sp. nov., left male palp. **A.** Prolateral; **B.** Ventral; **C.** Retrolateral. Abbreviations: C = conductor, E = embolus, ptA = proximal tibial apophysis, RTA = retrolateral tibial apophysis, TA = tegular apophysis, TAP I–III = projection I to III of tegular apophysis. Scale bars: 0.5 mm.

The male of *Oliosuniprocessus* Hu, Zhang & Liu, sp. nov. resembles that of *O.rossettii* (Leardi, 1901) (cf. Fig. 3A–C vs. figs 146–147 in Jäger 2020) by having two tibial apophyses, and small U-shaped embolus arising sub-centrally from the tegulum, but can be recognized by: 1) retrolateral tibial apophysis leaf-shaped in retrolateral view, with a serrated retrolateral margin; and 2) tegular apophysis complex, with a sharp projection (TAP I) prolaterally and a sharp projection (TAP III) on the embolic base (vs. retrolateral tibial apophysis triangular, tegular apophysis long triangular in *O.rossettii*).

##### Description.

**Male (holotype)**: Total length 9.0; DS 4.3 long, 4.2 wide; OS 4.8 long, 3.1 wide. Eyes: AME 0.31; ALE 0.22; PME 0.20; PLE 0.23; AME–AME 0.17; AME–ALE 0.06; PME–PME 0.31; PME–PLE 0.28; AME–PME 0.19; ALE–PLE 0.09; CHAME 0.17; CHALE 0.21. Measurements of palp and legs: Pp: 5.4 (1.7, 0.5, 1.2, -, 2.0); I: 23.0 (6.2, 2.1, 6.5, 6.4, 1.8); II: 26.4 (7.4, 2.0, 7.8, 7.3, 1.9); III: 17.8 (5.3, 1.7, 4.8, 4.5, 1.5); IV: 19.9 (5.9, 1.6, 5.3, 5.6, 1.5). Leg formula: II-I-IV-III. Spination: Pp: 131, 000, 3000; legs: Fe I 223, II–III 323, IV 220; Pa I–IV 000; Ti I 2134, II–III 2124, IV 2014; Mt I–III 2024, IV 3034. Cheliceral furrow with 2 anterior and 5 posterior teeth.

Palp (Fig. 3A–C): As in diagnosis, ptA arising submedially from Ti, RTA arising distally from Ti. Cymbium almost 1.5 times longer than Ti. TA complicated, with a sharp projection (TAP I) on prolateral margin, a lamellar projection (TAP II) on dorsal TA, and a sharp projection (TAP III) on embolic base. C tiny. E arising sub-centrally from tegulum in 10-o’clock-position.

**Figure 4.**
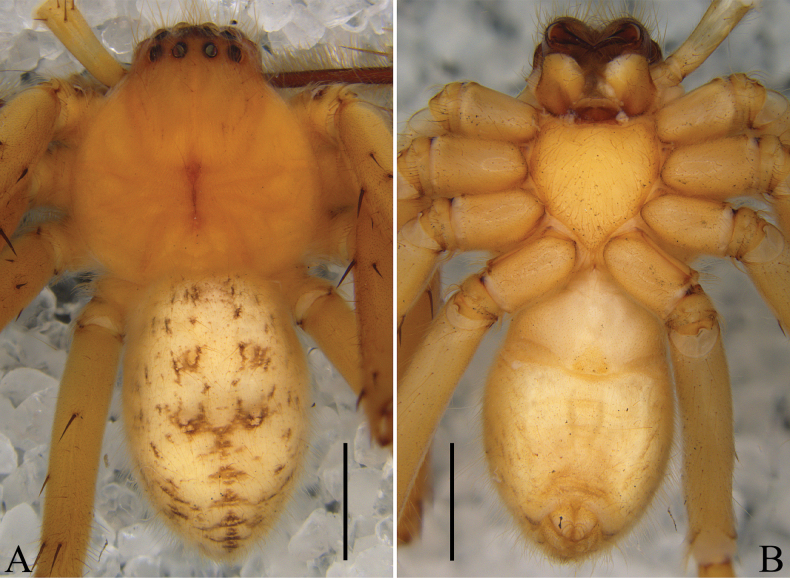
*Oliosuniprocessus* Hu, Zhang & Liu, sp. nov., male habitus. **A.** Dorsal; **B.** Ventral. Scale bars: 2 mm.

Colouration (Fig. 4A, B): DS orange, with thick hairs. Sternum and legs yellow. OS light yellow, dorsal with several brown marks.

**Female**: Unknown.

##### Distribution.

China (Chongqing Municipality) (Fig. 8).

***Olios* species without affiliation to a species-group**

**Remarks.** The following species do not exhibit clear affiliation with any of the species-groups proposed to date.

#### ﻿ Olios lincangensis

Taxon classificationAnimaliaAraneaeSparassidae

Hu, Zhang & Liu sp. nov.

A18223F2-3165-533A-AF95-F47F6A4C9CAF

https://zoobank.org/2F2871EE-9705-455D-8510-1B39551AEDE0

Figs 5 , 6 , 7 , 8

##### Type material.

***Holotype*** male: China • Yunnan Province: Lincang City, Yongde County, Daxueshan Yi, Lahu, Dai Township, Daxueshan Mountain, 24.11470°N, 99.64802°E, elevation 3428 m, 13 November 2021, Huachang Li leg. ***Paratype***: 1 female, with same data as for holotype.

##### Etymology.

The specific name is derived from the type locality: Lincang City; adjective.

##### Diagnosis.

**Figure 5.**
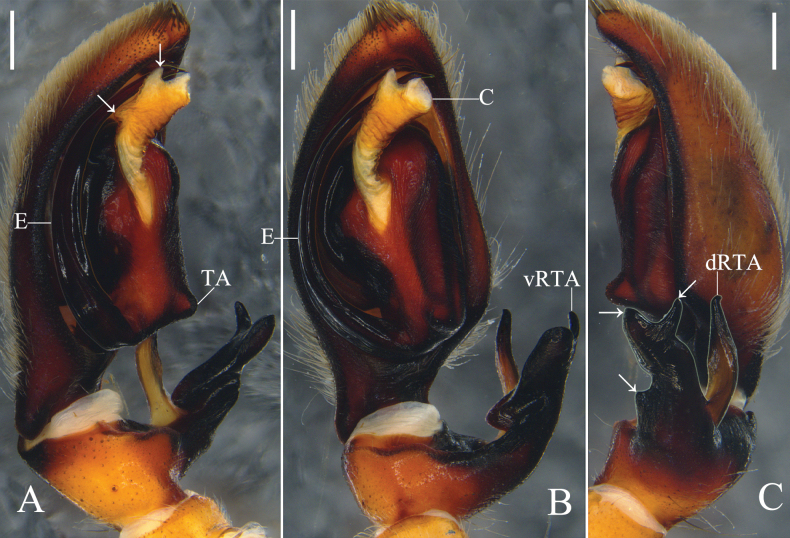
*Olioslincangensis* Hu, Zhang & Liu, sp. nov., left male palp. **A.** Prolateral, arrows point to the two apophysis of prolateral conductor; **B.** Ventral; **C.** Retrolateral, arrows point to the three apices of vRTA. Abbreviations: C = conductor, dRTA = dorsal part of retrolateral tibial apophysis, E = embolus, TA = tegular apophysis, vRTA = ventral part of retrolateral tibial apophysis. Scale bars: 0.5 mm.

The male of *Olioslincangensis* Hu, Zhang & Liu, sp. nov. resembles that of *O.feldmanni* Strand, 1915 (cf. Fig. 5A–C vs. figs 294–295 in Jäger 2020) by having a spike-like dorsal part of retrolateral tibial apophysis, complex ventral part of the retrolateral tibial apophysis, well-developed conductor arising sub-centrally from the tegulum in an 11-o’clock-position, and an embolus arising from the tegulum in a 6-o’clock-position, but can be recognized by: 1) main part of ventral part of retrolateral tibial apophysis irregularly thick forked, 2) tegulum with a small nipple-shaped tegular apophysis, and 3) conductor with two apophyses (see arrows in Fig. 5A) (vs. main part of ventral part of retrolateral tibial apophysis clavate, tegular apophysis absent, conductor without apophysis in *O.feldmanni*).

**Figure 6.**
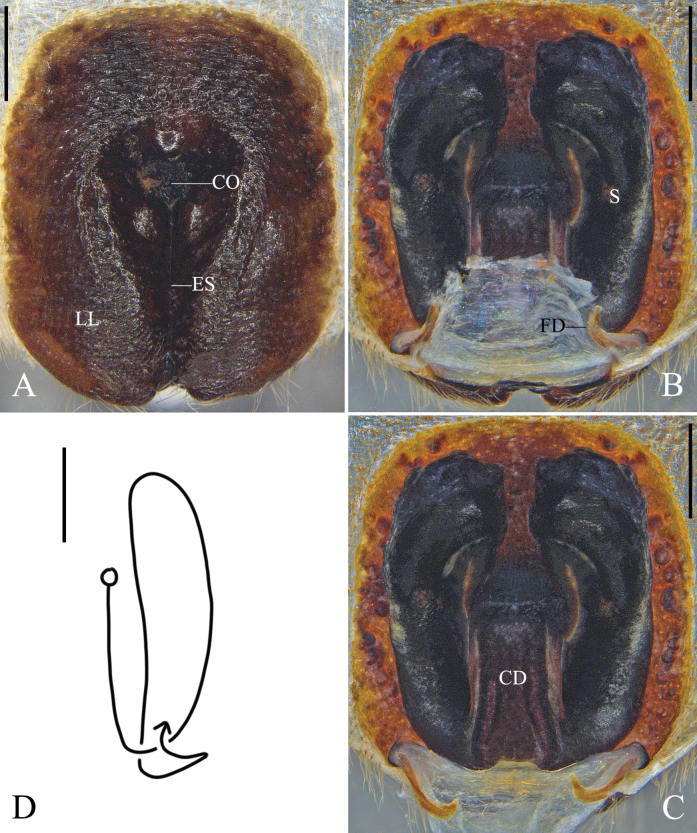
*Olioslincangensis* Hu, Zhang & Liu, sp. nov., female. **A.** Epigyne, ventral; **B.** Vulva, dorsal; **C.** Vulva, dorsal, the membrane between FD removed; **D.** Schematic course of right internal duct system, dorsal. Abbreviations: CD = copulatory duct, CO = copulatory opening, ES = epigynal slit, FD = fertilization duct, LL = lateral lobes, S = spermathecae. Scale bars: 0.5 mm.

The female of *Olioslincangensis* Hu, Zhang & Liu, sp. nov. resembles that of *O.croseiceps* (Pocock, 1898) (cf. Fig. 6A–D vs. figs 236–240 in Jäger 2020) by having an ungulate-shaped epigynal field, medially located copulatory ducts and large spermathecae, but can be recognized by the fertilization ducts laterally located, the distance between the basal fertilization ducts almost equal to the width of the epigynal field (vs. fertilization ducts medially located, the distance between basal fertilization ducts almost 1/3 of the width of the epigynal field in *O.croseiceps*).

##### Description.

**Male (holotype)**: Total length 12.5; DS 5.7 long, 5.8 wide; OS 7.1 long, 5.0 wide. Eyes: AME 0.31; ALE 0.33; PME 0.21; PLE 0.35; AME–AME 0.24; AME–ALE 0.29; PME–PME 0.54; PME–PLE 0.49; AME–PME 0.28; ALE–PLE 0.24; CHAME 0.17; CHALE 0.22. Measurements of palp and legs [I: missing]: Pp: 8.5 (2.6, 0.9, 1.4, -, 3.6); II: 28.0 (7.8, 2.2, 8.1, 7.7, 2.2); III: 21.3 (6.6, 2.0, 5.7, 5.2, 1.8); IV: 23.6 (7.1, 2.0, 6.4, 6.3, 1.8). Spination: Pp: 131, 000, 0001; legs: Fe II–IV 323; Pa II–IV 000; Ti II–IV 2124; Mt II–III 2024, IV 3025. Cheliceral furrow with 2 anterior and 5 posterior teeth.

Palp (Fig. 5A–C): As in diagnosis. Ti short, length of Ti almost 1/3 of cymbium. RTA well developed, vRTA with three apices in retrolateral view (see arrows in Fig. 5C). Tegulum with a small nipple-shaped TA. C tubular, with two aphophyses on prolateral part (see arrows in Fig. 5A). E thick and filiform.

**Figure 7.**
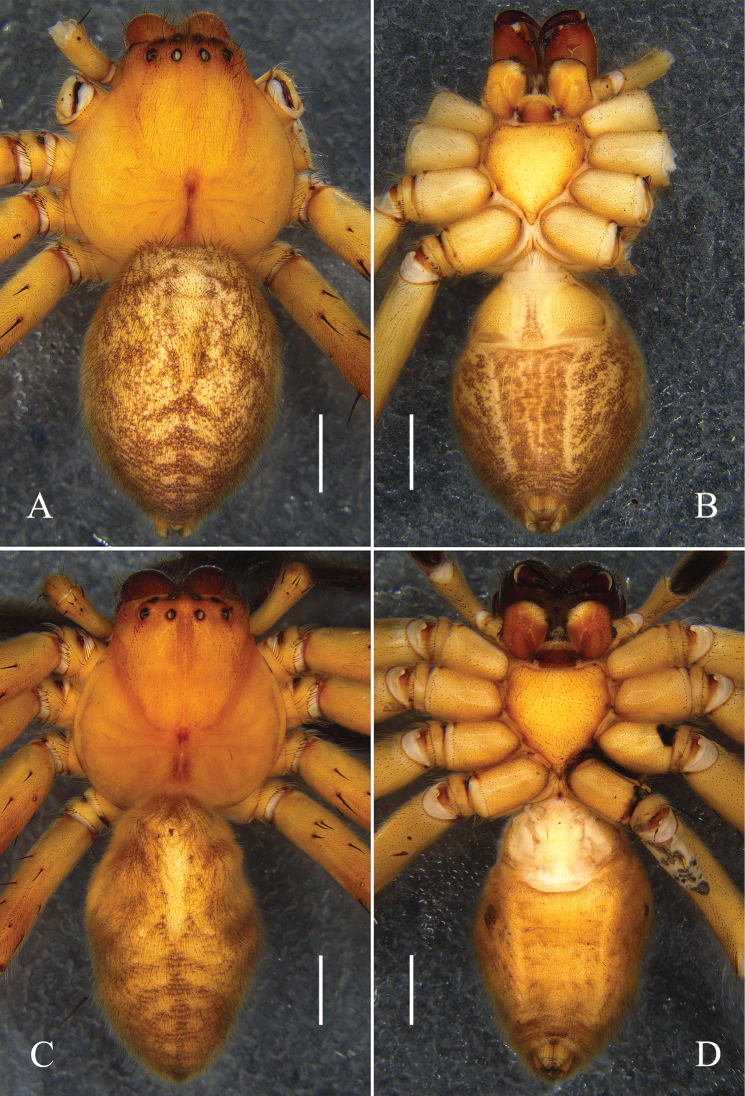
*Olioslincangensis* Hu, Zhang & Liu, sp. nov., habitus. **A, B.** Male (**A.** Dorsal; **B.** ventral); **C, D.** Female (**C.** Dorsal; **D.** Ventral). Scale bars: 2 mm.

Colouration (Fig. 7A, B): DS and sternum orange. Legs yellow to orange. OS dorsally yellow, with some brown marks, ventrally brown, with two yellow longitudinal lines.

**Female (paratype)**: Total length 14.1; DS 6.1 long, 6.7 wide; OS 8.3 long, 5.0 wide. Eyes: AME 0.35; ALE 0.32; PME 0.17; PLE 0.29; AME–AME 0.34; AME–ALE 0.29; PME–PME 0.67; PME–PLE 0.65; AME–PME 0.38; ALE–PLE 0.30; CHAME 0.14; CHALE 0.23. Measurements of palp and legs: Pp: 7.4 (2.2, 0.9, 1.5, -, 2.8); I: 23.5 (6.8, 2.3, 6.2, 6.2, 2.0); II: 25.9 (7.7, 2.4, 7.0, 6.8, 2.0); III: 18.7 (5.9, 1.8, 4.9, 4.4, 1.7); IV: 20.2 (6.1, 1.7, 5.3, 5.2, 1.9). Leg formula: II-I-IV-III. Spination: Pp: 131, 020, 1111, 1012; legs: Fe I 223, II–III 323, IV 321; Pa I–IV 000; Ti I–III 2024, IV 2004; Mt I–III 2024, IV 3034. Cheliceral furrow with 2 anterior and 5 posterior teeth.

Epigyne (Fig. 6A–D): As in diagnosis. Epigynal field rounded rectangular, longer than wide. Length of ES almost 2/3 of the length of epigynal field. Internal duct system obviously sclerotized. Length of CD almost half of the length of S. S large, with rough anterior part. FD long and narrow.

Colouration (Fig. 7C–D): As in male, but generally darker.

##### Distribution.

**Figure 8.**
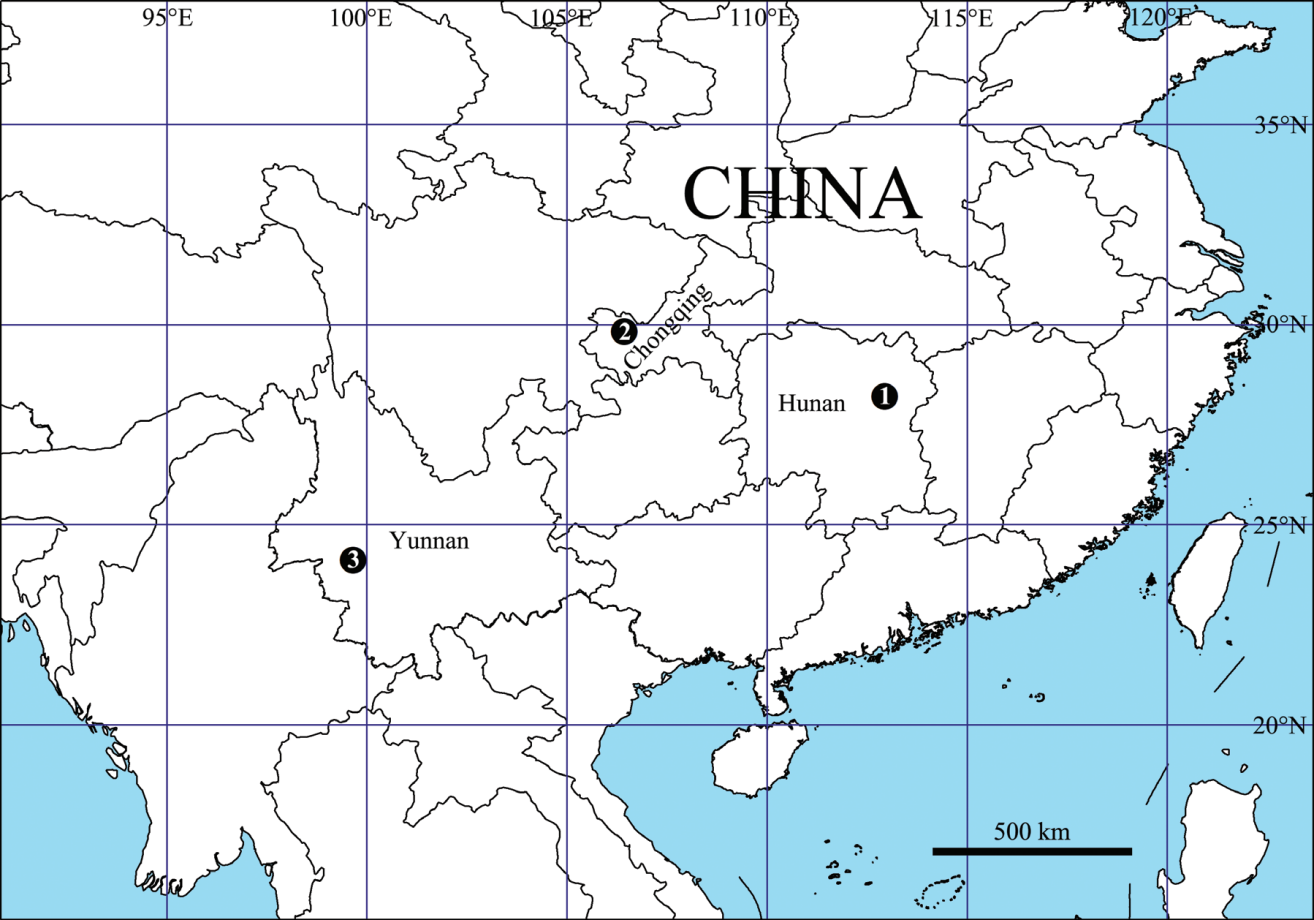
Distribution map of the three new *Olios* species 1 *O.biprocessus* Hu, Zhang & Liu, sp. nov. 2 *O.uniprocessus* Hu, Zhang & Liu, sp. nov. 3 *O.lincangensis* Hu, Zhang & Liu, sp. nov.

China (Yunnan Province) (Fig. 8).

## ﻿Discussion

The current paper reports three new *Olios* species from China: *O.biprocessus* Hu, Zhang & Liu, sp. nov., from Hunan Province; *O.uniprocessus* Hu, Zhang & Liu, sp. nov., from Chongqing Municipality; and *O.lincangensis* Hu, Zhang & Liu, sp. nov., from Yunnan Province. Two of these three new species (*O.biprocessus* and *O.uniprocessus*) belong to the *rossettii*-group, which is widely distributed across Asia and Africa (Jäger 2020). Jäger and Otto (2007) divided this species-group into two sub-groups based on the morphology of male palp: sub-group A with a simple RTA, sub-group B with both an RTA and a ptA. However, the position of the subtegulum in these sub-groups is inconsistent. Therefore, we consider proposing a new division of the sub-groups based on the position of subtegulum: group A with the subtegulum being located at a 6 o’clock position of the tegulum [e.g. *O.japonicus* Jäger & Ono, 2000, *O.kiranae* Sethi & Tikader, 1988, *O.kolosvaryi* (Caporiacco, 1947), *O.mahabangkawitus* Barrion & Litsinger, 1995, *O.sericeus* (Kroneberg, 1875), *O.suavis* (O. Pickard-Cambridge, 1876)]; group B with the subtegulum being invisible in ventral view [e.g. *O.biprocessus* Hu, Zhang & Liu, sp. nov., *O.rossettii* (Leardi, 1901), *O.tiantongensis* (Zhang & Kim, 1996), and *O.uniprocessus* Hu, Zhang & Liu, sp. nov.] (Jäger and Ono 2000; Jäger et al. 2002; Jäger and Kunz 2005; Jäger et al. 2022; Hu et al. 2025).

The copulatory organs of *Olioslincangensis* Hu, Zhang & Liu, sp. nov. show strong similarity to those of *Oliosfeldmanni* Strand, 1915 from Cameroon and *O.croseiceps* (Pocock, 1898) from Malawi, including a spike-like dRTA, complex vRTA, similar elongations of the conductor and embolus in the male palp, and similarly shaped lateral lobes and internal duct system in females. These three species probably belong to the same undefined species-group. The similar palpal conformation, especially RTA of *O.feldmanni* and *O.lincangensis*, suggests that this undefined group may be closely related to *Curicaberis* Rheims, 2015 (Sparassinae) from North and Central America (Rheims 2015).

The discovery of these new species in our research further enriches the species diversity of the genus *Olios* in China and provides new information on its geographical distribution in Asia. However, the females of two of the three new species are unknown. The low population density and docile nature of *Olios* spiders likely make them difficult to collect, resulting in less research attention compared to other Chinese sparassid genera such as *Pseudopoda* Jäger, 2000 and *Sinopoda* Jäger, 1999 (Zhang et al. 2025).

## Supplementary Material

XML Treatment for Olios

XML Treatment for Olios biprocessus

XML Treatment for Olios uniprocessus

XML Treatment for Olios lincangensis

## ﻿Additional information

### Author ORCIDs

He Zhang https://orcid.org/0000-0002-1478-9837

Jie Liu https://orcid.org/0000-0001-7744-9744

Caijun Wang https://orcid.org/0009-0005-2613-8117

Changhao Hu https://orcid.org/0009-0007-5591-3121

### Data availability

All of the data that support the findings of this study are available in the main text.
